# The roles of contact conformity, temperature and displacement amplitude on the lubricated fretting wear of a steel-on-steel contact

**DOI:** 10.1098/rsos.150637

**Published:** 2016-10-19

**Authors:** A. R. Warmuth, W. Sun, P. H. Shipway

**Affiliations:** Faculty of Engineering, University Technology Centre in Gas Turbine Transmission Systems, University of Nottingham, Nottingham NG7 2RD, UK

**Keywords:** fretting wear, contact geometry, temperature, displacement amplitude, lubricant, wear mechanism

## Abstract

This paper investigates the effect of contact geometry, temperature and displacement amplitude on the fretting behaviour of an aero-turbo oil lubricated cylinder-on-flat contact. To be effective, the lubricant needed both to penetrate the contact and then offer protection. Lubricant penetration into the fretting contact is found to be controlled by two physical parameters, namely (i) the width of the contact that remains covered throughout the fretting test and (ii) the lubricant viscosity. The protection offered by the lubricant (assuming that it has successfully penetrated the contact) is influenced by four physical parameters, namely (i) lubricant viscosity, (ii) traverse velocity, (iii) nominal contact pressure, and (iv) chemical effects. The relationship between the three experimental parameters which were varied in the programme of work (temperature, fretting displacement and cylinder radius) and physical parameters which influence the protection offered by the lubricant film can be competing, and therefore complex wear behaviour is observed. The roles of the various parameters in controlling the wear behaviour are presented in a coherent physical framework.

## 1. Introduction

Fretting is defined as small amplitude oscillatory motion between bodies that are in contact. Fretting wear dominates when a contact is within the *gross-sliding* regime (displacement amplitudes up to around 300 µm), with fretting fatigue dominating in *partial slip* [1]. There is no universally accepted definition regarding the transition between fretting wear (in the gross-sliding regime) and reciprocating sliding wear; however, the damage mechanisms associated with fretting wear are distinct from those associated with sliding wear, because the magnitude of movement in fretting is very small in comparison with the size of the contact area, and, as such, debris development within the contact and its subsequent retention within or ejection from the contact greatly influences wear, both in magnitude and mechanism [2].

Lubrication is a common palliative in reducing friction and wear in a variety of contact types, and it is therefore not surprising that it has also been employed in attempts to reduce fretting wear. In addition to its role as a lubricating fluid, the lubricant will restrict oxygen access into the fretting contact that will in turn affect the development of the oxide debris bed that is commonly associated with fretting wear. Moreover, owing to the very small and reciprocal motion associated with fretting, lubricant penetration into the contact may be poor [3]; it has been argued that this poor penetration is the cause of lubricated fretting contacts having been found to exhibit higher values of the coefficient of friction (COF) than observed in non-lubricated contacts [4]. A number of reports in the literature that address lubricated fretting wear have argued that wear behaviour is influenced predominately by the ability (or otherwise) of the lubricant and oxygen to penetrate the contact [5,6].

Liquid lubrication has been reported to be the most effective form of lubricant in reducing wear in gross-sliding fretting contacts [6]. Publications by both Halliday [7] (with silicone oils) and Shima *et al*. [4] (using polybutene oils) describe fretting experiments, using different grades of the lubricants across a range of viscosities. Both found that the rate of fretting wear was smaller with lubricants with lower viscosity, with this effect being attributed to the lubricant being able to more effectively penetrate the fretting contact as its viscosity was reduced. Shima *et al*. [4] also conducted fretting wear tests (over a range of displacement amplitudes) with different lubrication states as follows: without lubricant, with a low viscosity lubricant and with a high viscosity lubricant. They found that for tests conducted with very small displacement amplitudes (in the stick–slip regime), the COF was independent of the lubrication state, and proposed that this resulted from the fact that the majority of the contact was stuck, limiting the opportunity for the lubricant to penetrate that part of the contact. As the fretting displacement increased, the COF became more dependent upon the lubrication state, because more of the contact was slipping that facilitated lubricant penetration. At small displacement amplitudes within the gross-sliding regime, the COF for tests with the high viscosity lubricant was larger than for that observed in unlubricated tests. Both research teams observed that for a fixed fretting displacement amplitude, the wear rate was independent of lubricant viscosity as lubricant viscosity was increased up to a critical value, above which the wear rate rapidly increased [4,7]. It was concluded that this effect was associated not only with reduced lubricant penetration into the contact with increasing viscosity, but also with an associated reduction in effective transport of oxygen into the contact. It was proposed that this reduced oxygen transport would then limit the development of any protective oxide bed forming in the fretting contact.

Other research papers have also highlighted the link between fretting damage and the level of oxygen penetration into a lubricated contact (rather than just focusing on lubricant penetration itself) [8,9]. Wright [8] found that the concentration of oxygen in air was around six times that in a lubricant and furthermore argued that the rate of oxygen diffusion within the lubricant would be approximately proportional to the inverse of its viscosity. Research on fretting with semi-solid lubricants such as grease has indicated that the grease very effectively limits oxygen penetration into the contact, resulting in less oxide debris formation during fretting [5,10]; in similar work, higher rates of wear were observed in fretting contacts lubricated with a grease that had a ‘higher consistency’ [11]. Wang *et al*. [10] argued that the efficacy of grease lubrication in reducing fretting wear is dependent on the displacement amplitude, in that under large displacement amplitudes, more shear of the grease itself results in the release of base oil, which then is able to penetrate the fretting contact more effectively.

Lubrication of fretting contacts has been observed to influence not only the wear rate and COF, but also the boundaries between different modes of fretting. Under lubrication with both oils [6] and greases [12], the contact regimes were found to shift in relation to those identified in experiments conducted under unlubricated conditions. Liu & Zhou [6] demonstrated that the transition from both partial slip to mixed fretting and from mixed fretting to gross slip in lubricated contacts occurred at *higher* displacement amplitudes than under equivalent conditions in unlubricated contacts, and they argued that this shift of the transitions to higher displacement amplitudes resulted from (i) the inability of the lubricant to effectively penetrate the contact and (ii) the lubricant also limiting oxygen penetration into the contact. Together, these result in the contact being more metallic in nature, and thus more adhesive; accordingly, higher applied displacements are required to force the contact into the gross-slip regime [6]. Moreover, the displacements associated with the fretting regime boundaries increased with increasing lubricant viscosity [6].

Narayanan *et al*. [13] have investigated the influence of temperature on lubricated fretting wear of tin-plated copper contacts; again, it was proposed that the lubricant eliminated oxygen from the contact, although in this case, this was found to lead to a reduction in wear. They also observed a higher wear rate for lubricated tests at elevated temperatures, but attributed this to thermal softening of the tin coating. Other studies by McDowell [14], Neyman [15], Neyman & Sikora [11] have also concluded that the wear of a lubricated fretting contact is primarily influenced by the ability of the lubricant to exclude oxygen from the contact, and hence the reduced tendency to create brittle oxidized wear particles which thus results in a reduction in wear rate. The same conclusion, namely that oxygen exclusion would reduce fretting wear, was also reached by Shima *et al*. [4], but they suggested that this reduction was only observed in cases where the lubricant had been able to penetrate the contact; as such, the effects were most strongly observed with low viscosity lubricants in contacts fretting under low loads and high displacements.

In efforts to better understand lubricant penetration, Imai *et al*. [16] conducted research on flat-on-flat fretting contacts and studied the ability of grooves to channel oil into the centre of the contact (the geometry of flat-on-flat contacts more effectively restricts oxygen and lubricant penetration than point or line contact geometries which are more commonly employed in experimental work). Fretting wear damage was significant on surfaces without grooves and was reduced substantially when grooves were introduced [16]. Different groove configurations were examined, and it was shown that the reduction in wear rate increased as the spacing between the grooves decreased (i.e. as the lubricant was more effectively transported into the contact) [16].

Most experimental research on fretting is conducted using simplified contact geometries (such as point contact or line contact). Wear rates derived from such experiments have then been used in the prediction of wear in more complex contacts based upon the assumption that the wear rate is independent of the contact geometry itself [17]. However, recent research has cast doubt on the validity of this assumption. Fouvry *et al*. [18] and Merhej & Fouvry [19] have demonstrated that in unlubricated non-conforming contacts (cylinder-on-flat and ball-on-flat, respectively), the fretting wear rate was dependent on the radius of curvature and decreased as the radius of curvature was increased. It was proposed that this decrease in wear rate was due to the reduced tendency for the debris to be ejected from more-conforming contacts, with the entrapped debris protecting the contact from further wear. More recently, Warmuth *et al*. [20,21] have made similar observations (again, with an unlubricated cylinder-on-flat contact geometry) and argued that the reduction in wear rate with increasing contact conformity is due to a change in wear mechanism. In a contact with low conformity (namely, a smaller cylinder radius), oxygen was able to penetrate the contact readily, facilitating the formation of oxide wear debris which then flowed out of the contact. In contrast, as the contact became more-conforming (larger cylinder radius), oxygen was more effectively excluded from the centre of the contact, resulting in metal–metal adhesion and metal transfer between the contact faces; at high fretting displacement amplitudes, this transfer was significant and the formation of substantial pits and peaks within the contact (with features measuring up to 140 µm deep) was observed. Fouvry & Merhej [22] also found that for large radius specimens (i.e. a more-conforming contact), a metallic region in the centre of the contact developed and this was also attributed to effective oxygen exclusion.

In the light of the strong influence of contact conformity on the rates and mechanisms of fretting wear in unlubricated contacts and the general understanding that rates and mechanisms of wear in lubricated fretting contacts are influenced both by lubricant penetration into the contact and the effect of the lubricant in limiting oxygen into the contact, this study examines the effect of contact conformity in lubricated fretting wear. As part of this study, the effect of temperature and displacement amplitude on the rates and mechanisms of wear across is also addressed (noting that temperature markedly influences the lubricant properties). All experiments were conducted in a static oil bath, using a fully formulated, high-performance, synthetic ester-based turbo oil which is commonly used in aeroengine applications.

## 2. Experimental procedure

### 2.1. Materials, test conditions and procedures

Fretting wear experiments were conducted on a high strength steel (specification BS S132). Heat treatment of the steel was conducted prior to machining of the specimens to size (the details of this heat treatment process can be found elsewhere [23]) and resulted in the specimens having a hardness (*HV*_20_) of 465–480 kgf mm^−2^. A high thermal stability aviation turbo oil (BP2197) was used as the lubricant throughout this work; BP2197 contains tricresyl phosphate (TCP) as an extreme pressure (EP) additive.

**Figure 1.**
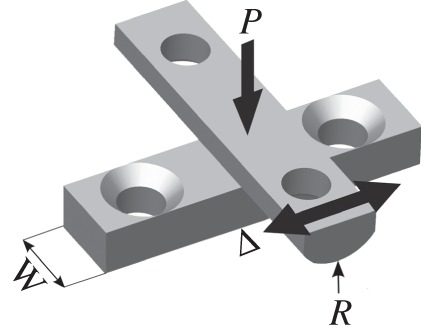
Crossed cylinder-on-flat specimen configuration used in fretting tests.

**Figure 2.**
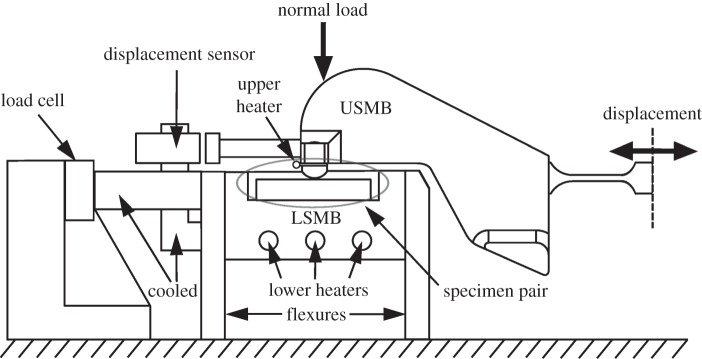
Illustration of the main components of the fretting apparatus used in this study.

The specimen pair was assembled in a cylinder-on-flat configuration, as shown in figure 1. The flat and cylindrical specimens were ground on a linear and cylindrical grinder, respectively. The flat and cylindrical specimens had a roughness (Ra) of 0.1 to 0.3 µm and 0.4 to 0.7 µm, respectively. The flat specimen is mounted on the lower specimen mounting block (LSMB) which is stationary and the cylindrical specimen is mounted on the upper specimen mounting block (USMB). Before the specimens were brought into contact, the surrounding oil bath was filled with approximately 20 ml of oil, so that the contact was submerged with the oil level being approximately 1 mm above the contact region. The USMB was loaded through a dead weight configuration, with the normal load that results being termed *P*. During assembly of the fretting pair, the USMB is able to rotate around the axis of fretting; application of a small load to the pair at this stage ensures that the axis of the cylindrical specimen lies in the plane of the flat specimen, whereupon the rotation of the USMB is fixed. The main components in the rig used for the fretting experiments are illustrated in figure 2. Both the USMB and the LSMB are heated via cartridge heaters. The motion of the USMB (and hence the cylindrical specimen) is created by a force generated by an electromagnetic vibrator (EMV). The motion of the USMB (and hence the cylindrical specimen) is created by a sinusoidally varying force generated by an EMV. The displacement, Δ, of the USMB is monitored by a capacitance displacement sensor which is mounted to the LSMB and is recorded throughout the duration of the test. The amplitude of the force input is controlled to achieve a set displacement amplitude, Δ*, for the test, although (owing to compliances in the system) the displacement–time profile is not sinusoidal.

The lateral force, *Q*, is measured (and recorded) throughout the test by a piezoelectric load cell which is connected to the quasi-stationary LSMB. To protect the force and displacement sensors, water cooled members separate them from the heated area of the rig as shown in figure 2. Both displacement and load sensors have been calibrated (both externally and *in situ*) in static conditions. The load and displacement signals are sampled at a rate of two hundred measurements per fretting cycle for all of the experiments.

**Figure 3.**
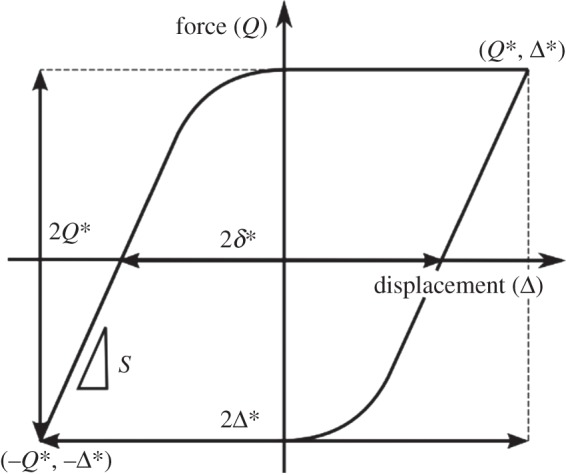
Schematic diagram of an ideal fretting loop in the gross-sliding regime, illustrating parameters defined in the text.

The behaviour of the contact can be monitored throughout the test by examination of the fretting loops; an idealized gross-slip loop is plotted in figure 3. The displacement of the USMB is measured, but it must be noted that this is not the same as the slip in the contact; there is compliance in the system which physically separates the contact from the point of measurement, and hence the measured displacement amplitude, Δ*, is always slightly larger than the contact slip amplitude, δ*. The actual contact slip amplitude (δ*) can be derived by measuring the displacement at zero force, as indicated in figure 3.

The COF is defined as follows:

2.1 $$\text{COF}=\frac{Q^{*}}{P}.$$

Cylinder-on-flat fretting tests were conducted with cylinders with radii, *R*, of 6, 15, 80 and 160 mm; the *least-conforming* contact pairs used cylinders with a radius of 6 mm and the *most-conforming* contact pairs used cylinders with a 160 mm radius. The flat specimen has a width, *W*, of 10 mm as seen in figure 1 (this defines the contact length). Experiments were conducted under normal loads, *P*, of 250 and 650 N and applied displacement amplitudes, Δ*, of 25, 100 and 300 µm. The far-field surface temperature of each specimen was measured via a thermocouple that was spot welded to both specimen surfaces on the centreline and at a distance of approximately 10 mm from the fretting contact; these thermocouples were used for independent control of the upper and lower heaters (in all tests, the upper and lower specimens were set to the same temperature); the lubricant itself was not directly heated. Insulation of the whole working area during testing results in all of the main components being at the same temperature, including the bulk of the lubricant bath; it is, however, recognized that flash temperatures in the contact will result in thermal excursions in the area of the contact. Experiments were conducted with the lubricant at ambient temperature (typically 22°C), 85, 150 and 230°C and run at a frequency of 20 Hz for a duration of 100 000 cycles; test information is summarized in table 1. To explore the repeatability of behaviour (while conducting the programme of work in an efficient manner), tests under selected parameters from the test matrix were repeated. In these cases, wear volumes varied by no more than 10%, and similar evolution of COF was observed.

**Table 1.** Summary of the fretting test parameters.

lubricant temperatures	ambient, 85, 150 and 230°C
cylinder radii, *R*	6, 15, 80 and 160 mm
displacement amplitudes, Δ*	25, 100 and 300 µm
test duration, *C*	100 000 cycles
normal load, *P*	250 and 650 N
oscillation frequency	20 Hz

### 2.2. Estimation of wear volume and surface topography

**Figure 4.**
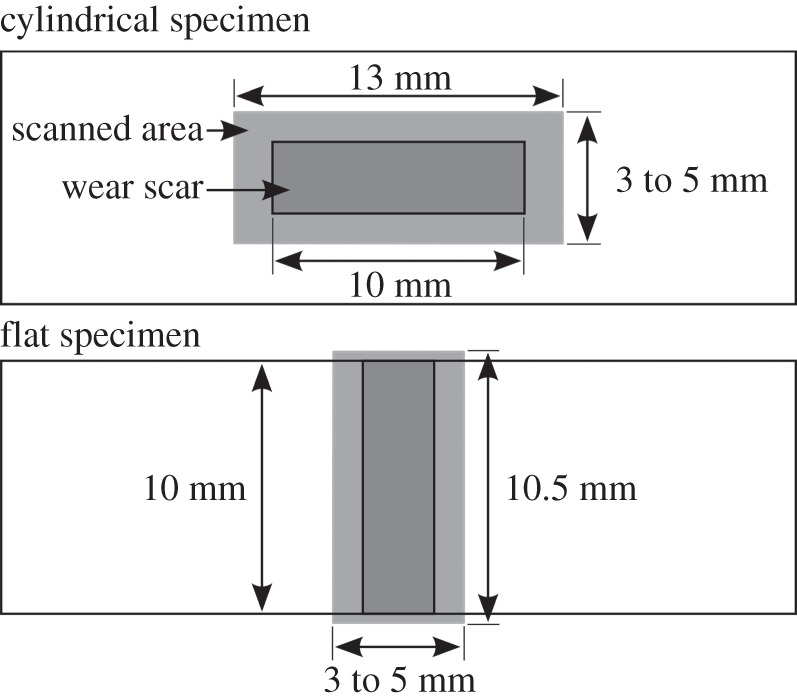
Illustration of the area measured by profilometry with respect to the fretting wear scar.

**Figure 5.**
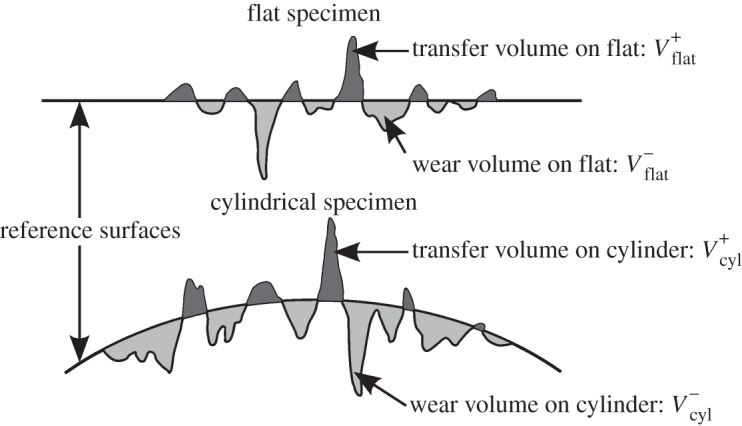
Illustration of the definition of wear and transfer volumes in a fretting scar for both the flat and cylindrical specimens.

After completion of a fretting experiment, specimens were lightly swabbed with industrial methylated spirit to remove loose debris and residual oil, thus leaving any debris that was adhered to the specimen in place. To evaluate their topography, the wear scars on both the flat and cylindrical specimens were scanned using a Bruker contour GT-I interferometer, which has a vertical resolution of approximately 0*.*15 nm and a lateral resolution of 4 µm. The scan area was always larger than the wear scar (figure 4) to allow the reference surface (representing the surface before wear occurred) to be defined by interpolation from the surfaces outside the wear scars (as proposed by Elleuch & Fouvry [24] and illustrated in figure 5). The volume below each reference surface was regarded as the wear volume ($V_{\mathrm{Flat}}^{-}$ and $V_{\mathrm{Cyl}}^{-}$ for the flat and cylindrical specimens, respectively) and the volume of material above the reference surfaces was regarded as the transferred volume ($V_{\mathrm{Flat}}^{+}$ and $V_{\mathrm{Cyl}}^{+}$ for the flat and cylindrical specimens, respectively), although it is recognized that this may be displaced metal or oxide debris, and may originate from either specimen. The total wear and transfer volumes for the couple (*V*^−^ and *V*^+^, respectively) are defined as the sum of the respective volumes for the flat and cylindrical specimens. Wear and transfer rates for the contact pair were calculated based on the average test slip amplitude (δ*) and the number of cycles per test (C), as defined in equation (2.2):

2.2 $$\begin{array}{rl} {\dot{V}}^{-} & =\frac{V^{-}}{4\delta^{*}\text{PC}}\\ \;\text{and}\;{\dot{V}}^{+} & =\frac{V^{+}}{4\delta^{*}\text{PC}}. \end{array}\}$$

### 2.3. Characterization of wear scars and debris

Scanning electron microscopy (SEM) was used to characterize the nature of the wear scars, using a Philips XL30 SEM. Back scattered electron (BSE) images were used to distinguish oxide from metallic material (oxide which forms in the wear scar has a lower average atomic number, resulting in a lower brightness in BSE imaging than the steel). The identification of oxide was confirmed qualitatively by energy-dispersive X-ray spectroscopy (EDX) analysis.

### 2.4. Covered contact width and lubricant viscosity

In this experimental programme, the vast majority of tests were conducted across a range of contact geometries with an applied load of 250 N. Assuming a Young's modulus of 207 GPa and a Poisson's ratio of 0.28 for this steel [25], Hertzian contact mechanics for a line contact was used to predict the contact semi-width (*b*) and initial maximum Hertzian contact pressure (*σ*_H,max_). Values of *b* and *σ*_H,max_ are presented in table 2 for the four contact geometries employed.

**Table 2.** Hertzian contact semi-width, initial maximum Hertzian contact pressure and lower-bound estimate of the covered semi-widths for the contact geometries and displacement amplitudes examined (*P* = 250 N).

			Δ* (μm)		
			25	100	300
radius (mm)	*b* (μm)	*σ*_H, max_ (MPa)	2 *b_c_* (μm)		
6	41	386	32	0	—
15	65	244	80	—	—
80	151	106	252	—	—
160	213	75	376	226	0

**Figure 6.**
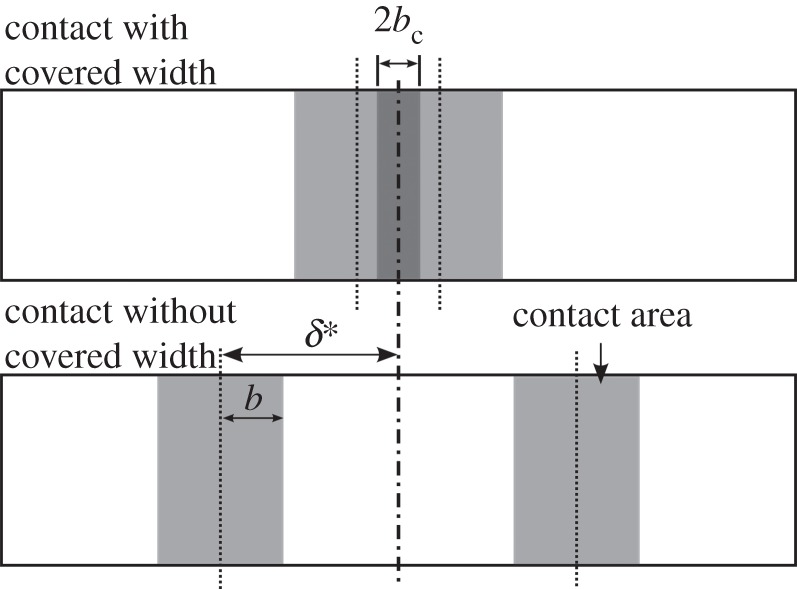
Illustration of contacts over a cycle of motion with covered width (top) and without covered width (bottom).

As the displacement amplitudes that are being studied are comparable in size to the elastic contact width, under certain conditions there exists a portion that remains covered throughout the duration of the test as illustrated in figure 6 and described by equation (2.3):

2.3 $$b_{c}=\left\{ \begin{array}{ll} 0, & b<\delta^{*}\\ (b-\delta^{*}), & b\geq \delta^{*}. \end{array}\right.$$

A number of the terms are variable throughout the test, and, as such, *b*_c_ cannot readily be defined for a given test set-up. In a situation where the displacement amplitude (Δ*) is controlled, the slip amplitude in a cycle (δ*) is dependent upon the tractional force in that cycle as follows [26]:

2.4 $$\delta^{*}=\Delta^{*}-\frac{Q^{*}}{S},$$

where *S* is the contact stiffness, as indicated in figure 3. Results presented later in this paper indicate that with an applied load of 250 N, the highest value of COF observed is approximately 0.65; this results in a maximum value of Q* of approximately 160 N. Analysis of the fretting loops for the contacts in this paper indicates that the contact stiffness is approximately 38 N µm^−1^ [27]; accordingly, the difference between the applied displacement amplitude (Δ*) and the slip amplitude (δ*) would be no more than 4.3 µm, and will be smaller for smaller values of COF. In addition, owing to wear of the contact during the test, the actual contact width will grow from its initial (Hertzian) size of *b*.

As such, the values of the covered semi-width, *b*_c_, throughout the test cannot be precisely determined, but can be estimated via equation (2.3) if (owing to their similarity) δ* can be assumed to be equal to Δ* and it is assumed that the contact width can be assumed to be the initial Hertzian contact width (*b*). With these assumptions, the estimate of *b*_c_ will always be a lower bound (because the actual contact width will always be greater than the Hertzian contact width and the slip amplitude will always be less than the displacement amplitude). Estimated lower-bound values of the covered widths for the different combinations of cylinder radii and Δ* examined in this work are presented in table 2.

The literature indicates that lubricant viscosity has a significant influence on the behaviour of a lubricated fretting contact (as outlined in §1), with the lubricant viscosity itself being strongly dependent upon temperature. The viscosities of the turbo oil employed (BP 2197) at −40°C and 100°C were supplied by the manufacturer [28], and from these, the viscosity at other temperatures can be estimated [29]. The measured and estimated values of viscosity as a function of temperature are presented in table 3.

**Table 3.** Estimates of the viscosity of the lubricant at the test temperatures alongside those provided by the manufacturer.

temperature (°C)	kinematic viscosity (10^−3^ Pa s)
−40	12500
22	59.6
85	7.21
100	5.28
150	2.45
230	1.16

## 3. Experimental results

### 3.1. Characteristics of the fretting motion

**Figure 7.**
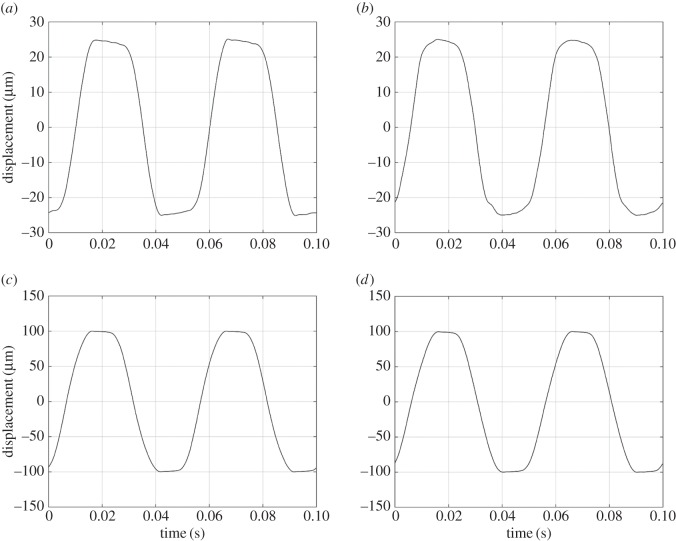
Applied displacement as a function of time for lubricated contacts under ambient conditions and an applied load of 250 N: (*a*) *R* = 6 mm and Δ* = 25 µm; (*b*) *R* = 160 mm and Δ* = 25 µm; (*c*) *R* = 6 mm and Δ* = 100 µm; (*d*) *R* = 160 mm and Δ* = 100 µm. All data are taken from approximately midway through the fretting test at around 50 000 cycles.

The fretting motion is delivered via the EMV. Examples of displacement–time loops (figure 7) indicate that in each case, there is a period where the contact is essentially stationary (the small slopes of the curves in these regions are associated with system compliance), followed by a period of gross-sliding (where the slope is much larger in magnitude). For the cases where Δ* = 25 µm, the speed in the sliding period is approximately constant for the vast majority of the period of slip, with a value of approximately 5 mm s^−1^. When Δ* = 100 µm, the speed in sliding is slightly higher for the case when *R* = 6 mm (19 mm s^−1^) than it is when *R* = 160 mm (15 mm s^−1^).

**Figure 8.**
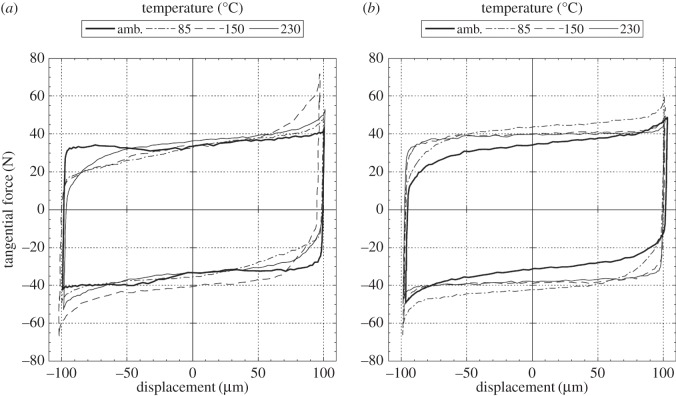
Fretting loops at 99 000 cycles for the lubricated fretting tests conducted at temperatures of ambient, 85, 150 and 230°C, with a 100 µm applied displacement amplitude: (*a*) *R* = 6 mm; (*b*) *R* = 160 mm.

The fretting characteristics are commonly represented by fretting loops, examples of which are presented in figure 8. Each of these loops represents behaviour very close to the end of the respective experiment (following 99 000 fretting cycles). It can be seen that the behaviour is similar with both contact conformities; variations of the force across the sliding regions of the loops can be seen which are generally attributed to the development of geometrical features associated with wear [26,30,31]. In addition, the steep sides of the loops represent the contact stiffness, with a value of approximately 38 N µm^−1^ being observed irrespective of the lubricant temperature and contact conformity.

### 3.2. Effect of contact conformity

**Figure 9.**
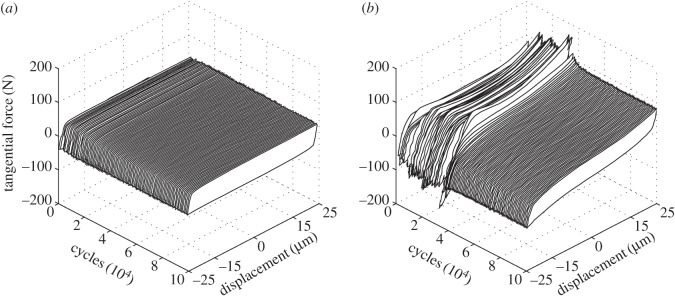
Time progression of fretting loops for lubricated fretting tests conducted at ambient temperature with a 25 µm displacement amplitude and a 250 N normal load: (*a*) *R* = 6 mm; (*b*) *R* = 160 mm.

Experiments were conducted with cylinders of different radii (6, 15, 80 and 160 mm) at ambient temperature and with a 25 µm displacement amplitude. Figure 9 shows the evolution of the shape of the fretting loops with number of cycles through the tests for both the 6 mm and the 160 mm radii cylinders. It can be seen that the loops from the least-conforming contact do not change significantly throughout the test, whereas those from the most conforming contact indicate unsteady behaviour in the initial stages (with higher tractional forces) which then settles to more steady behaviour after approximately 350 000 cycles.

**Figure 10.**
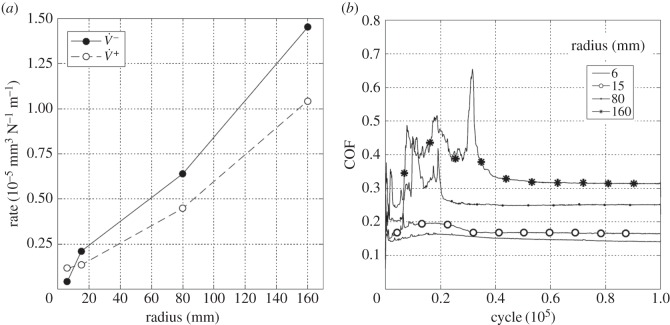
(*a*) Plot of wear rate (${\dot{\text{V}}}^{-}$) and transfer rate (${\dot{\text{V}}}^{+}$) as a function of cylinder radius and (*b*) plots of development of COF with number of fretting cycles. Experiments used cylinders with radii of 6, 15, 80 and 160 mm and were conducted at ambient temperature with Δ* = 25 µm and *P* = 250 N.

Figure 10*a* is a plot of the wear and transfer rates for the experiments conducted with different contact radii. The wear and transfer rate for the least-conforming contact (6 mm cylinder radius) was very low, with rates of less than 0.1 × 10^−5^ mm^3^ N^−1^ m^−1^. The wear and transfer rates increased as the contacts became more conforming, and were around 15 times larger for the contacts with the 160 mm cylinder radius in comparison with those with the 6 mm cylinder radius. The difference between the wear and transfer rates also became larger with increasing cylinder radius, indicating that material loss from the contact was increasing.

Figure 10*b* shows the COF over the duration of the test for the different contact conformities examined. The couples with 6 and 15 mm cylinders exhibited low values of COF with little change over the test duration; in both cases, a small initial rise was observed (with some instabilities in this region) which then subsequently fell to a steady value. In contrast to these, the tests with the 80 and 160 mm radius cylinders initially exhibited high and unstable values of COF over extended numbers of cycles. The period of instability, and the magnitude of the instability both increased as the conformity of the contact increased. In addition, the steady-state COF also increased with increasing contact conformity.

**Figure 11.**
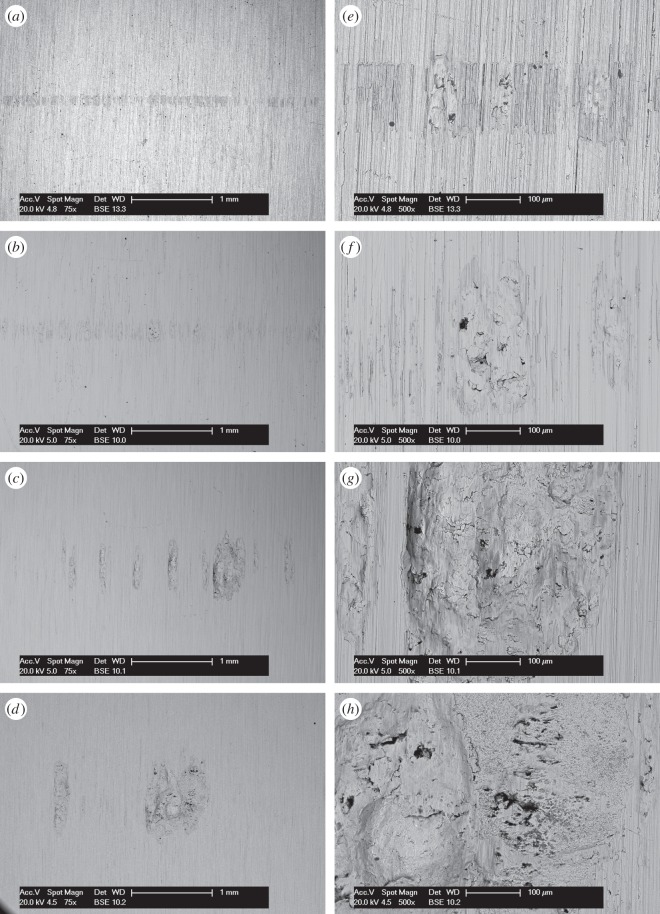
(*a*--*h*) BSE images of the top surface of the wear scars on the flat specimens at low and high magnification following experiments conducted on cylinders with radii of (*a*, *e*) 6, (*b*, *f*) 15, (*c*, *g*) 80 and (*d*, *h*) 160 mm, at ambient temperature with Δ* = 25 µm and *P* = 250 N.

BSE images of the fretting wear scars on the flat specimens are presented in figure 11 for the experiments conducted with different contact conformities (figure 11*a–d* at low magnification and figure 11*e–h* at higher magnification). Images of the fretting wear scar from the contact with the 6 mm radius cylinder show that the scar is patchy and damage appears to be in the form of scratches in regions that were high points within the contact. For the tests with 15 mm radius cylinders, the scratches become less pronounced and areas of plasticity and material transfer can be observed in the centre of the contact. Despite the nominal contact pressure reducing with increasing cylinder radius (table 2), the wear scars from the tests with the 80 and 160 mm radius cylinders exhibit many much larger areas of plasticity and material transfer. All the damaged regions are clearly primarily metallic, because the contrast associated with oxide-based debris beds when observed in BSE imaging is absent. However, some very dark spots are observed in the damaged regions which are shown to be carbon-rich.

**Figure 12.**
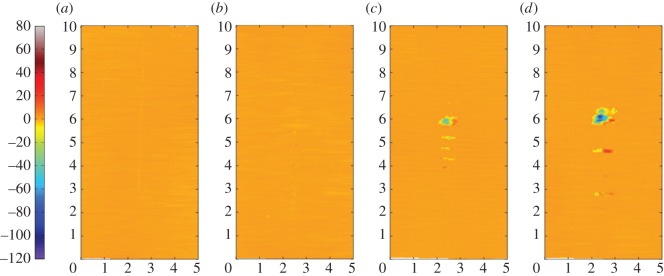
(*a*--*d*) Topography of the surface of the flat specimen for the experiments conducted on cylinders with radii of (*a*) 6, (*b*) 15, (*c*) 80 and (*d*) 160 mm, at ambient temperature with Δ* = 25 µm and *P* = 250 N. The dimensions on each profile are in millimetres; the colour scale on the left is in micrometres and relates to the height of the surface.

Figure 12*a–d* are the corresponding profilometric contour plots of the surfaces of the fretting wear scars on the same flat specimens. The wear scars for the tests with the 6 mm radius cylinder and the 15 mm radius cylinder extend across the entire width of the specimen; however, in both cases, the damage is very shallow (typically of the same order of magnitude as the surface roughness). In contrast, damage to the surface of the flat following fretting against a cylinder with an 80 mm radius is dominated by a few large deep pits, with one as deep as 50 µm. For the wear scar in the most conforming fretting contact (160 mm radius cylinder), the pits are both deeper and wider than those observed in the contact with the 80 mm cylinder, but are again isolated and few in number; the largest pit exhibits a maximum depth of 95 µm.

### 3.3. Fretting behaviour of the least-conforming contacts in lubricated fretting

#### 3.3.1. Effect of lubricant temperature with small fretting amplitude

**Figure 13.**
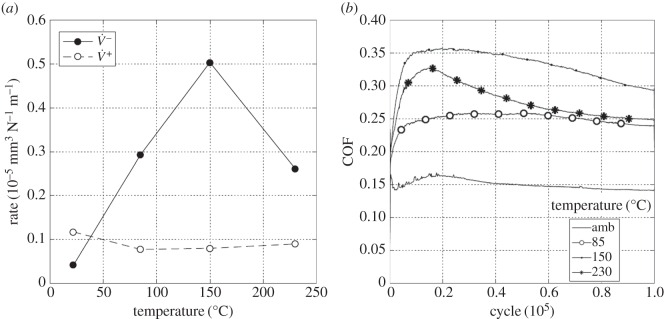
(*a*) Plot of wear rate (${\dot{\text{V}}}^{-}$) and transfer rate (${\dot{\text{V}}}^{+}$) as a function of test temperature and (*b*) plots of development of COF with number of fretting cycles. Experiments used 6 mm cylinders and were conducted at ambient temperature, 85, 150 and 230°C with Δ* = 25 µm and *P* = 250 N.

Experiments were conducted with the least-conforming contact geometry (6 mm radius cylinders) with a 25 µm displacement amplitude and with a range of lubricant temperatures (ambient, 85, 150 and 230°C). Figure 13*a* presents the wear and transfer rates for the experiments conducted at different test temperatures. It can be seen that at ambient temperature, both the wear and the transfer rates were very low. As the temperature was increased to 150°C, the wear rate increased dramatically (to more than 10 times its value at ambient temperature), but the transfer rate changed very little. However, the wear rate for the test conducted at 230°C was almost half of that observed in the test conducted at 150°C (again, there was little change in the transfer rate).

Figure 13*b* shows the evolution of the COF throughout the test as a function of test temperature. The COF was observed to increase with increasing test temperature up to 150°C, with the COF in the test conducted at 150°C being around twice that observed in the ambient temperature test. On further increasing the test temperature to 230°C, the COF rose rapidly to a maximum value of 0.33, and then dropped (again, relatively rapidly) to a value of 0.25.

**Figure 14.**
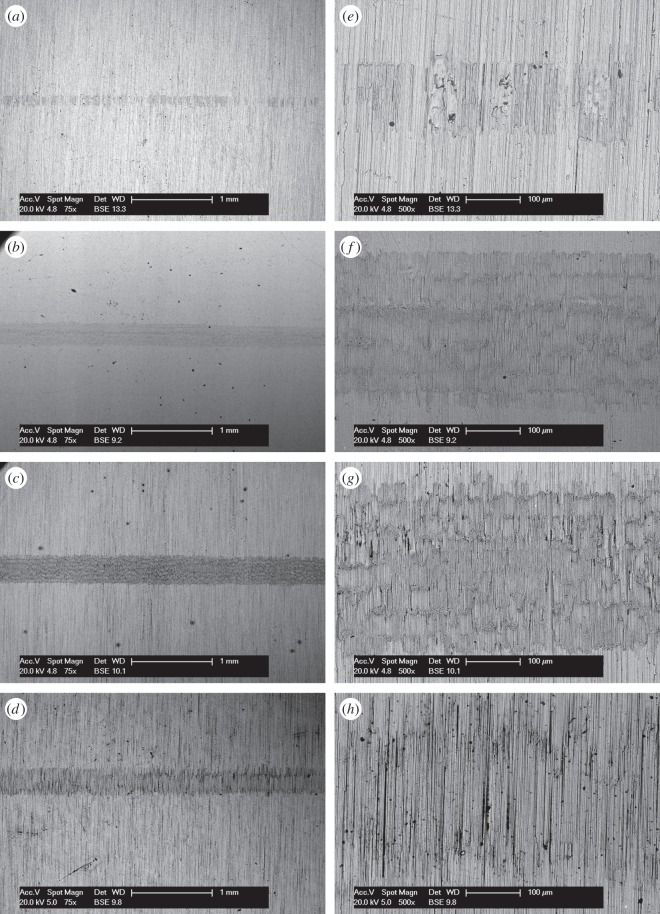
(*a*--*h*) BSE images of the top surface of the wear scars on the flat specimens at low and high magnification following experiments with 6 mm cylinders conducted at (*a*, *e*) ambient, (*b*, *f*) 85, (*c*, *g*) 150 and (*d*, *h*) 230°C, with Δ* = 25 µm and *P* = 250 N.

BSE images of the fretting wear scars on the flat specimens from the experiments conducted with the least-conforming contact at different lubricant temperature are presented in figure 14 (figure 14*a–d* at low magnification and figure 14*e–h* at higher magnification). The surface of the specimen from the test conducted with lubricant at ambient temperature exhibits scratching behaviour with little sign of surface plasticity; damage appears to be occurring only at the high points within the contact, leaving other areas undisturbed. At higher lubricant temperatures (85 and 150°C), wave-like prows were observed to form on the surface; these were most pronounced in the experiment conducted with a lubricant temperature of 150°C. As the lubricant temperature was further increased to 230°C, these prow formations were no longer in evidence, with the surface exhibiting significant scratching instead.

**Figure 15.**
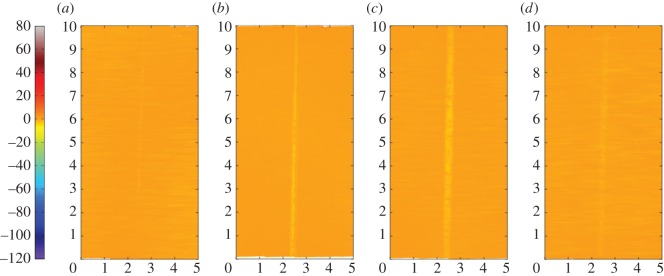
(*a*--*d*) Topography of the surface of the flat specimen following experiments with 6 mm cylinders conducted at (*a*) ambient, (*b*) 85, (*c*) 150 and (*d*) 230°C, with Δ* = 25 µm and *P* = 250 N. The dimensions on each profile are in millimetres; the colour scale on the left is in micrometres and relates to the height of the surface.

Figure 15*a–d* are the corresponding profilometric contour plots of the surfaces of the flat specimen fretting wear scars. It can be seen that all of the wear scars extend across the full width of the contact. The wear scar from the test conducted at ambient temperature exhibits very little surface damage, it being less than 1 µm deep. Following testing at 85 and 150°C, the wear scars became wider and deeper, having a depth of around 3 and 4 µm, respectively. However, following testing at 230°C, the resulting wear scar was shallower again, having a depth of only 2 µm (although its width was similar to that of the scar developed following testing at 150°C).

#### 3.3.2. Effect of lubricant temperature with higher fretting amplitude

**Figure 16.**
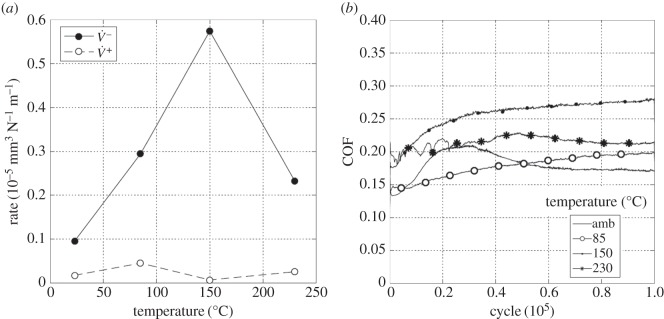
(*a*) Plot of wear rate (${\dot{\text{V}}}^{-}$) and transfer rate (${\dot{\text{V}}}^{+}$) as a function of test temperature and (*b*) plots of development of COF with number of fretting cycles. Experiments used 6 mm cylinders and were conducted at ambient temperature, 85, 150 and 230°C with Δ* = 100 µm and *P* = 250 N.

To compare directly with the results presented in §3.3.1, experiments were conducted with the least-conforming contact geometry (with 6 mm radius cylinders) with lubricant temperatures of ambient, 85, 150 and 230°C and a higher displacement amplitude of 100 µm. Figure 16*a* is a plot of the resulting wear and transfer rates. By comparing with figure 13*a*, it can be seen that the rates of wear follow the same pattern (with very similar values) to those derived from tests conducted at the lower displacement amplitude of 25 µm. The transfer rate is, however, slightly lower at the higher displacement amplitude, and again remains low across the range of test temperatures examined. Figure 16*b* shows the development of COF over the whole duration of the tests conducted with the different lubricant temperatures with the larger (100 µm) displacement amplitude. Again, by comparing with figure 13*b*, similar trends can be seen, although the final values of the COF are different at the two displacement amplitudes. Notably, at both displacement amplitudes, the COF was smallest for the test conducted at ambient temperature, and largest for the test conducted at 150°C.

**Figure 17.**
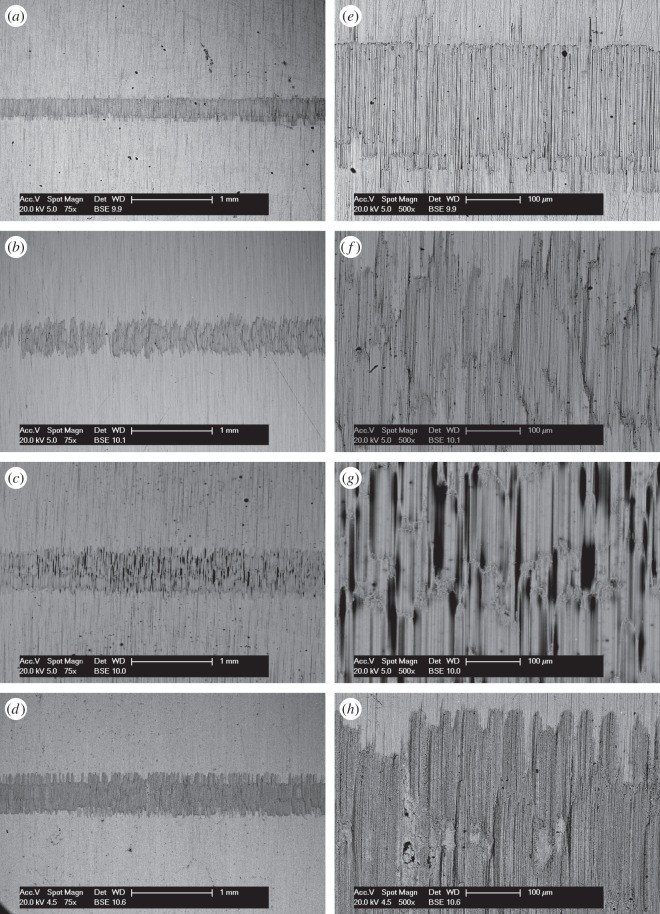
(*a*--*h*) BSE images of the top surface of the wear scars on the flat specimens at low and high magnification following experiments with 6 mm cylinders conducted at (*a*, *e*) ambient, (*b*, *f*) 85, (*c*, *g*) 150 and (*d*, *h*) 230°C, with Δ* = 100 µm and *P* = 250 N.

BSE images of the fretting wear scars on the flat specimens for the experiments conducted at different temperatures with the larger 100 µm displacement amplitude are shown in figure 17 (figure 17*a–d* are at low magnification and figure 17*e–h* are at higher magnification). The surface of the specimen fretted at ambient temperature exhibits scratching behaviour similar to that observed following testing at the smaller 25 µm displacement amplitude (figure 14); however, the scratches are deeper and occur across the entire contact width. Following fretting at 85 and 150°C, the scratches became more pronounced and prows developed, with the degree of surface disruption increasing with increasing temperature. In a similar manner to the tests conducted at the smaller displacement amplitude, the wear scar following testing at 230°C exhibited significant damage; however, this was less significant than that following testing at 150°C.

**Figure 18.**
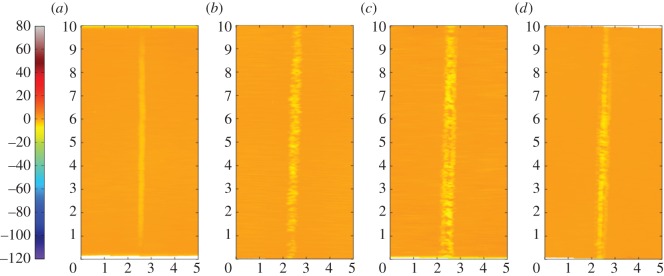
(*a*--*d*) Topography of the surface of the flat specimen following experiments with 6 mm cylinders conducted at (*a*) ambient, (*b*) 85, (*c*) 150 and (*d*) 230°C, with Δ* = 100 µm and *P* = 250 N. The dimensions on each profile are in millimetres; the colour scale on the left is in micrometres and relates to the height of the surface.

Figure 18*a–d* show the corresponding profilometric contour plots of the fretting wear scars on the surfaces of the flat specimens at the different test temperatures. It can be seen that all of the wear scars extend across the width of the contact. The specimen from the test conducted at ambient temperature exhibited the least damage, with the scar being only approximately 4 µm deep. The wear scars following tests at 85 and 150°C were 8 and 10 µm deep, respectively; however, as the test temperature was further increased to 230°C, the depth of the wear scar decreased to 7 µm.

### 3.4. Fretting behaviour of the most-conforming contacts in lubricated fretting

#### 3.4.1. Effect of lubricant temperature with lower fretting amplitude

**Figure 19.**
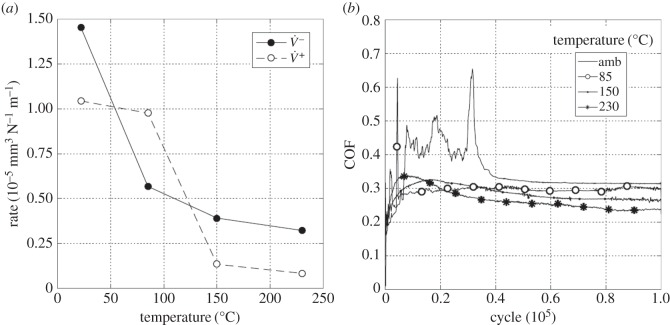
(*a*) Plot of wear rate (${\dot{\text{V}}}^{-}$) and transfer rate (${\dot{\text{V}}}^{+}$) as a function of test temperature and (*b*) plots of development of COF with number of fretting cycles. Experiments used 160 mm cylinders and were conducted at ambient temperature, 85, 150 and 230°C with Δ* = 25 µm and *P* = 250 N.

Experiments were conducted with the most conforming contact geometry (using 160 mm radius cylinders) over a range of test temperatures (ambient, 85, 150 and 230°C) with a 25 µm displacement amplitude. Figure 19*a* shows the wear and transfer rates as a function of test temperature; the pattern of behaviour is very different from that observed for the tests with the least-conforming contacts (figures 13*a* and 16*a*). At ambient temperature, both the wear and the transfer rates were very high, the wear rate being 1.45 × 10^−5^ mm^3^ N^−1^ m^−1^. As the test temperature was increased, the wear rate decreased monotonically and significantly, with the wear rate at 230°C being approximately one-fifth of that observed in the ambient temperature test. Again, in contrast to the tests with the least-conforming contacts, the transfer rate closely followed the wear rate, becoming lower as the test temperature was increased.

Figure 19*b* shows the evolution of COF for the tests conducted at the different temperatures. The COF for the ambient temperature test was initially low, but rose very rapidly and became unstable, reaching a maximum COF of 0.65; however, after around 40 000 cycles, this instability disappeared and the COF gradually decreased to a steady value of around 0.31. The test conducted at 85°C again exhibited instability (and high values) of the COF in the very early stages of the test; however, in this case the COF settled to a steady value after only around 6000 cycles. At the two higher test temperatures (150 and 230°C), the early instability in the COF observed at the lower test temperatures was absent. In contrast to the behaviour observed for the least-conforming contact, it is observed that the steady-state values of the COF decreased with increasing test temperature.

**Figure 20.**
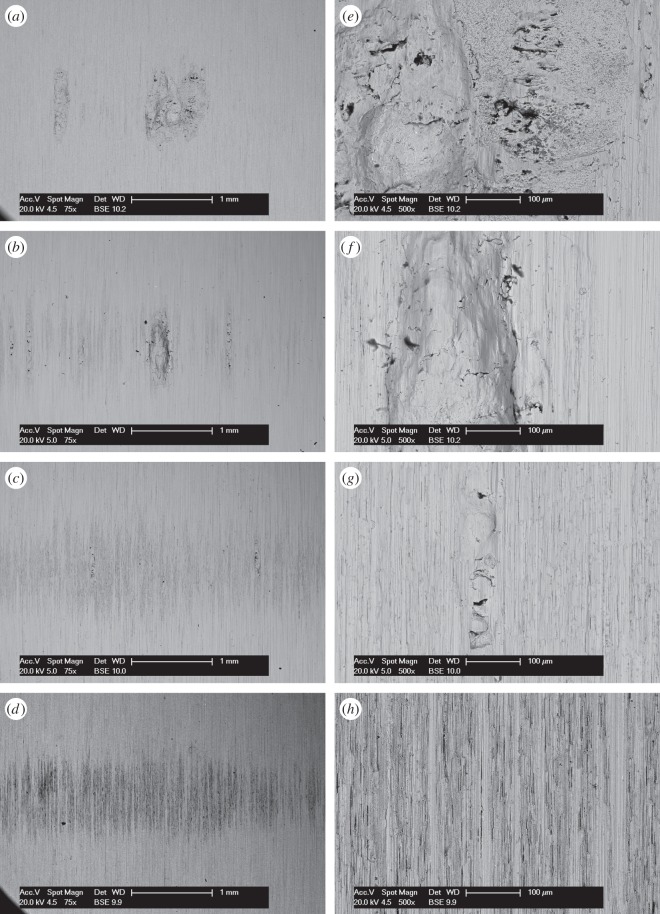
(*a*--*h*) BSE images of the top surface of the wear scars on the flat specimens at low and high magnification following experiments with 160 mm cylinders conducted at (*a*, *e*) ambient, (*b*, *f*) 85, (*c*, *g*) 150 and (*d*, *h*) 230°C, with Δ* = 25 µm and *P* = 250 N.

**Figure 21.**
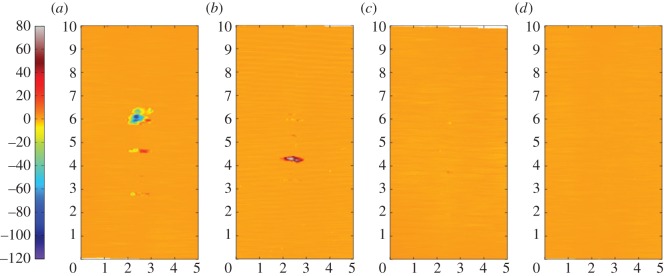
(*a*--*d*) Topography of the surface of the flat specimen following experiments with 160 mm cylinders conducted at (*a*) ambient, (*b*) 85, (*c*) 150 and (*d*) 230°C, with Δ* = 25 µm and *P* = 250 N. The dimensions on each profile are in millimetres; the colour scale on the left is in micrometres and relates to the height of the surface.

BSE images of the fretting wear scars on the flat specimens for the most-conforming contact experiments with different lubricant temperatures and a 25 µm displacement amplitude are shown in figure 20 (figure 20*a–d* are at low magnification and figure 20*e–h* are at higher magnification). The surface following the test at ambient temperature exhibits significant material transfer, most of it occurring in a large consolidated region in the centre of the contact. Following testing at 85°C, dark scratches become evident and the transfer became more concentrated to just the centre of the contact. Following testing at 150°C, the transfer regions are almost completely eliminated and the scratches become more apparent. Finally, following testing at 230°C, the surface does not show any evidence of material transfer, with only dark scratches being evident. Figure 21*a–d* are the corresponding profilometric contour plots of the surface of the fretting wear scars on the flat specimens following testing at the different temperatures. The ambient temperature test exhibits the most damage, with a very wide and deep pit in the centre of the contact (95 µm deep). Following testing at 85°C, the wear scar exhibits a narrow, but large peak of material (approx. 80 µm high) in the centre of the contact. In contrast, the wear scars following testing at 150 and 230°C exhibit very little notable damage, indicating that the scratches and surface damage are very shallow.

#### 3.4.2. Effect of lubricant temperature with higher fretting amplitude

**Figure 22.**
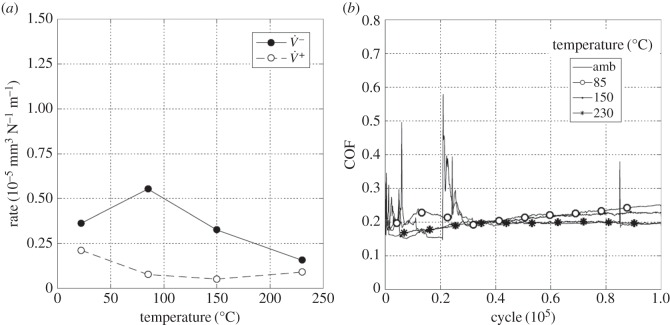
(*a*) Plot of wear rate (${\dot{\text{V}}}^{-}$) and transfer rate (${\dot{\text{V}}}^{+}$) as a function of test temperature and (*b*) plots of development of COF with number of fretting cycles. Experiments used 160 mm cylinders and were conducted at ambient temperature, 85, 150 and 230°C with Δ* = 100 µm and *P* = 250 N.

Experiments were conducted with 160 mm radius cylinders, at temperatures of ambient, 85, 150 and 230°C, at the higher (100 µm) displacement amplitude. Figure 22*a* is a plot of wear and transfer rates for the different test temperatures. It can be seen that the wear and transfer rates are much smaller than the corresponding rates at the smaller displacement amplitude (figure 19*a*). In general terms, as the test temperature is increased, the wear and transfer rates decrease; for example, at 230°C, the wear rate is nearly half of that at ambient temperature.

Figure 22*b* shows the evolution of the COF for the tests conducted at the different test temperatures at the higher displacement amplitude of 100 µm. As was observed for the lower displacement tests with this contact conformity (figure 19*b*), the COF for the tests conducted at both ambient temperature and at 85°C exhibited instability in the early stages of the test. However, with the higher displacement amplitude, some small instability was observed in the test conducted at 150°C which was not observed in the test conducted at the lower displacement amplitude. In addition, a very brief period with a higher COF of approximately 0.35 was observed at the beginning of the test conducted at the highest temperature, before it settled to a steady (and low) value. By comparing with figure 19*b*, it can be seen that the increased displacement amplitude has resulted in a reduction in steady-state COF across all test temperatures.

**Figure 23.**
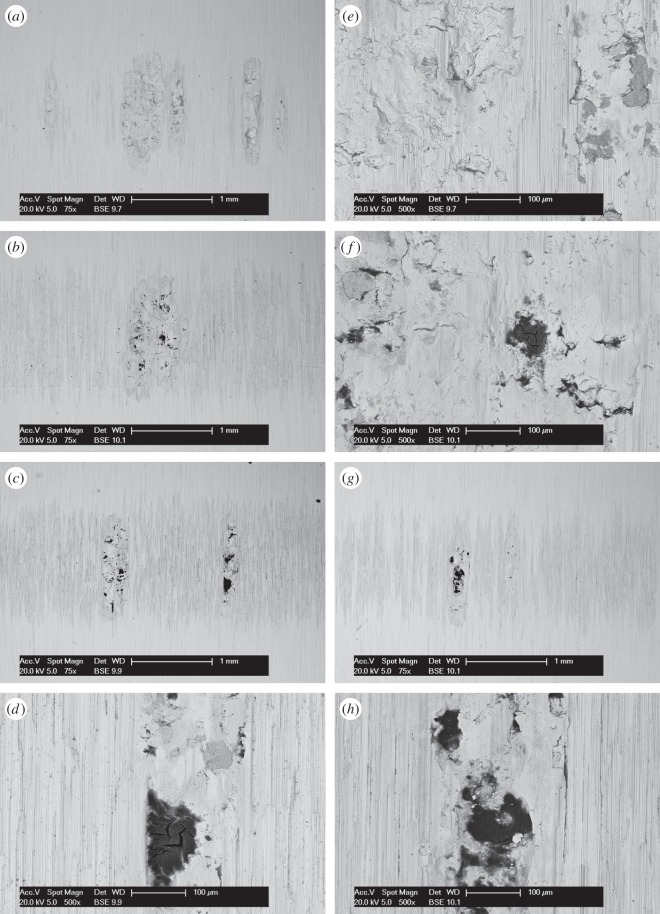
(*a*--*h*) BSE images of the top surface of the wear scars on the flat specimens at low and high magnification following experiments with 160 mm cylinders conducted at (*a*, *e*) ambient, (*b*, *f*) 85, (*c*, *g*) 150 and (*d*, *h*) 230°C, with Δ* = 100 µm and *P* = 250 N.

**Figure 24.**
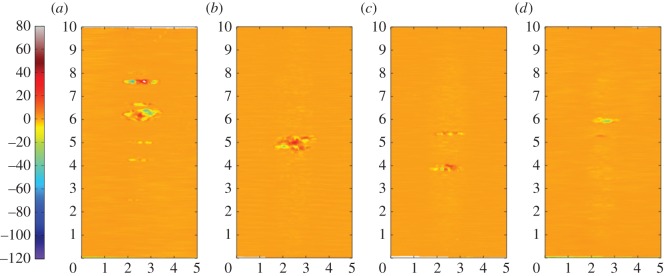
(*a*--*d*) Topography of the surface of the flat specimen following experiments with 160 mm cylinders conducted at (*a*) ambient, (*b*) 85, (*c*) 150 and (*d*) 230°C, with Δ* = 100 µm and *P* = 250 N. The dimensions on each profile are in millimetres; the colour scale on the left is in micrometres and relates to the height of the surface.

BSE images of the fretting wear scars on the flat specimens from experiments with the most-conforming contacts and the 100 µm displacement amplitude as a function of lubricant temperature are shown in figure 23 (figure 23*a–d* are at low magnification and figure 23*e–h* are at higher magnification). The specimen surface following testing at ambient temperature exhibits significant material transfer, with the majority occurring in the centre of the contact. These regions of transfer are very large, and other regions on the contact show only small scratches. As the test temperature was increased, the degree of material transfer decreased; however, it is notable that significant material transfer did take place across the range of test temperatures examined. In addition, all the areas of material transfer exhibited regions that appeared dark in BSE imaging; these regions were qualitatively shown to have a very high carbon content by EDX analysis. Figure 24*a–d* are the corresponding contour plots where the scale of the material transfer can be observed in all cases. The ambient temperature test exhibited the most surface damage in the form of very wide pits and peaks, with some pits being as deep as approximately 50 µm.

## 4. Discussion

**Figure 25.**
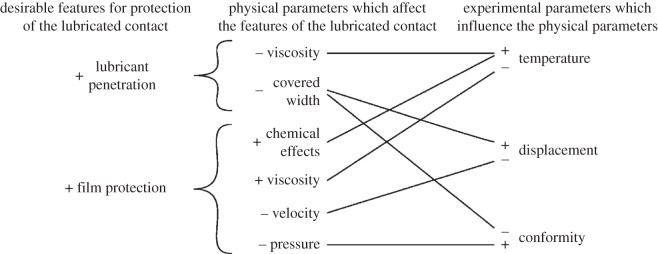
Interaction diagram, linking the required elements of protection to the experimental parameters investigated in this study through the physical parameters which are influenced by the experimental parameters. The symbols (+) and (−) represent higher and lower values respectively, and lines indicate the linking trends (e.g. a higher displacement amplitude will result in a lower covered width, whereas a lower displacement amplitude will result in a lower contact velocity).

It will be argued in the forthcoming discussion that lubricated fretting wear behaviour is strongly controlled by (i) the ability of the lubricant to penetrate fretting contact and (ii) the ability of any lubricant that has penetrated the contact to protect the surfaces from fretting damage. As such, behaviour in lubricated fretting is strongly linked to the contact geometry and lubricant temperature. Lubricant penetration into fretting contacts has previously been studied using lubricants with different viscosities [4–7] where it was found that fretting damage was limited for low viscosity lubricants and increased as the lubricant viscosity increased. This study has also demonstrated that lubricant viscosity strongly influences fretting damage, although in this work, variations in viscosity were produced through changes in temperature (it is argued that this is more representative of many fretting contacts where the temperature does vary with time, but it is also recognized that changes in temperature may affect other parameters associated with fretting in addition to the lubricant viscosity). This work has also demonstrated that the use of a lubricant effectively limits oxygen penetration into the fretting contact; this reduced oxygen level can result in severe adhesive transfer during fretting if the lubricant has not itself been able to protect the contact. This finding is in accord with the work of Shima *et al*. [4], but suggests that oxygen exclusion from a fretting contact is only beneficial in reducing fretting wear if another mechanism (as opposed to the formation of an oxide debris bed) is in place to limit adhesive transfer. For all of the experiments conducted, it was found that there was a strong link between surface disruption and damage in the fretting contact and the highest values of the COF; it is concluded that the majority of surface damage occurs in the early stages of the test, when the COF tended to exhibit any instabilities and higher values. An interaction diagram (figure 25) has been developed to relate the features required for effective protection of the fretting contact by the lubricant (namely (i) lubricant penetration into the contact and (ii) film protection by any lubricant which has penetrated) to the experimental parameters investigated in this study (temperature, displacement amplitude and contact conformity); this is achieved through a basic physical understanding of how the experimental parameters influence the physical parameters that control the behaviour of the lubricated contact. A more detailed discussion is now presented which provides the background to these more general descriptions of behaviour.

### 4.1. Covered width

It is proposed that significant fretting wear damage occurs in lubricated conditions if there is poor penetration of lubricant into the contact. Lubricant penetration into a fretting contact for this research has been found to be fundamentally related to lubricant viscosity and contact covered width. Owing to the small displacement amplitudes associated with fretting, under certain experimental conditions, a portion of the contact may remain covered throughout an entire fretting cycle. As a measure of this covered portion, the initial widths of the region not unexposed to open lubricant (2*b*_c_), have been calculated for all of the experiments conducted and are presented in table 2. It must be noted that these are based upon the initial Hertzian contact, and, as wear of the contact occurs, the covered width will increase.

Results from experiments conducted on contacts over the range of conformity at low displacement amplitude and at ambient temperature (where the lubricant viscosity is highest and therefore its penetration into the contact least favoured; figure 10*a*) indicate that wear and transfer rates for tests conducted with the most conforming contacts (160 mm) were around 15 times larger than those with the least-conforming contacts (6 mm). This increase in damage is due to the most-conforming contacts being able to exclude lubricant and oxygen from the centre of the contact. As oxygen is effectively limited in this region, a layer of oxidized debris (normally observed to be protective in fretting in unlubricated conditions) cannot form, and because there is no fluid film separating the surfaces, severe adhesive transfer occurs. For all of the tests conducted at ambient temperature with different conformities (different cylinder radii), the wear rate was only slightly higher than the transfer rate. This indicates that what is being removed from one surface is largely being transferred to the other, but both wear and transfer being high indicate that the contact has suffered significant damage. Adhesive transfer is more evident as the contact becomes more conforming (because it better excludes lubricant from the contact) as shown in figure 11. As shown in figure 10*b*, all contact conformities examined resulted in an initial period of higher and less stable COF; for the most-conforming contact geometry, the COF was as high as 0.65. For the least-conforming contacts, it is proposed that the initial period with a slightly raised (and slightly less stable) COF is associated with the removal of high points on the contact; in contrast, the high and unstable COF for the more-conforming contacts is associated with material transfer. In these cases, the subsequent decrease in COF indicates that the lubricant was eventually able to more effectively enter the contact zone; it is proposed that the transferred material slightly separated the surfaces or that the roughening of the surface facilitated penetration of the lubricant into the contact.

**Figure 26.**
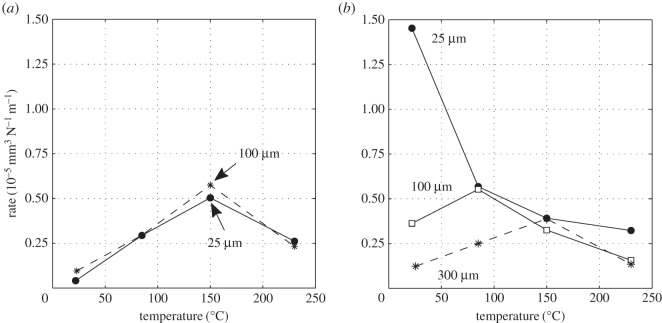
Plots of wear rate (${\dot{\text{V}}}^{-}$) as a function of test temperature for experiments conducted with *P* = 250 N at different displacement amplitudes for experiments using (*a*) 6 mm and (*b*) 160 mm cylinders.

Displacement amplitude can also affect lubricant penetration into the contact, beacause the covered width (2*b*_c_) decreases with increasing displacement amplitude. The wear behaviour in tests conducted at ambient temperature with 6 mm radius cylinders (which do not have a large initial covered width) was not strongly influenced by displacement amplitude (figure 26*a*). These tests exhibited wear rates of 0.04 × 10^−5^ and 0.1 × 10^−5^ mm^3^ N^−1^ m^−1^ for 25 µm and 100 µm displacement amplitudes, respectively. In contrast, the wear behaviour for experiments conducted at ambient temperature with the most conforming contacts (which have a large elastic covered width) were strongly dependent on displacement amplitude, with these tests exhibiting wear rates of 1.45 × 10^−5^ and 0.36 × 10^−5^ mm^3^ N^−1 ^m^−1^ for 25 and 100 µm displacement amplitudes, respectively (figure 26*b*). In this case, there was still a substantial initial covered width of 226 µm when fretted with the 100 µm displacement amplitude; to further examine the role of the initial covered width, additional experiments were conducted only for this contact geometry with a displacement amplitude of 300 µm, this value being selected because it was high enough to ensure that the initial covered width was zero. The wear rate for tests conducted with this larger displacement amplitude at ambient temperature resulted in a wear rate of 0.12 × 10^−5^ mm^3^ N^−1^ m^−1^ which was even lower than that observed in the experiment conducted with a displacement amplitude of 100 µm (figure 26*b*). This wear rate is almost as low as that observed in the experiments conducted with 6 mm cylinders at ambient temperature, with these very low rates being associated with the contacts being fully flooded with lubricant in both cases.

As discussed, at ambient temperature, the 6 mm contacts at both displacement amplitudes examined exhibit minimal damage, indicating that they are protected by a fluid film. The levels of damage are indicated by the low wear rates, with BSE images of the surfaces (figures 14*e* and 17*e*) also exhibiting little evidence of wear or material transfer. These images also show that the contact does not wear past that expected from the initial Hertzian contact semi-width. For the 6 mm contact under the 250 N load, the elastic semi-width is estimated to be 41 µm (table 2); when added to the displacement amplitude (values of 25 µm and 100 µm), the semi-width of contact interaction on the flat specimen initially should be 66 and 141 µm, respectively. As seen from figures 14*e* and 17*e*, scratches across the contact are very close to these initial interaction widths, suggesting that wear is only occurring at the high points on the contact.

### 4.2. Viscosity

Lubricant penetration into the contact can also be facilitated by a reduction in the lubricant viscosity. In these tests, a reduction in lubricant viscosity is associated with an increase in test temperature, although it is recognized that temperature may exert other influences on the behaviour of the contact in fretting. As lubricant has already been shown to effectively penetrate the least-conforming (6 mm) contact at ambient temperature, it is to be expected that an increase in the test temperature (and thus a reduction in lubricant viscosity) would not improve this lubricant penetration. However, for the most-conforming contacts, it has been shown that lubricant was not able to fully penetrate the contacts with the larger covered widths at ambient temperature, and thus it is to be expected that an increased test temperature (and thus reduced lubricant viscosity) will exert an influence here. The contacts that had the largest covered width (i.e. the most-conforming contact with a displacement amplitude of 25 µm) exhibited wear and transfer rates which were substantially higher when tests were conducted at ambient temperature than when conducted at 230°C (figure 19*a*). The transfer rate was very similar to the wear rate, indicating that material was not being lost, but simply transferred around the contact. The high initial COF (figure 19*b*) for the test conducted at ambient temperature is indicative of material transfer; as material transfer roughens the surface, the lubricant is eventually able to penetrate the contact region and both lower and stabilize the COF. At higher test temperatures, the lubricant is better able to penetrate the contact and prevent transfer and damage, with a more stable COF being observed. This hypothesis is supported by the BSE images of the contact (figure 20); at low temperatures, the contact exhibits significant levels of material transfer while at higher temperatures, the contact exhibits more evidence of scratching and less transfer, but with high points on the surface still resulting in significant damage to the contact.

Experiments conducted at ambient temperature with 160 mm cylinders at the higher displacement amplitudes of 100 and 300 µm exhibit wear rates that are substantially lower than those associated with the experiments performed with a 25 µm displacement amplitude (figure 26*b*). However, for all displacement amplitudes, the wear rates all tended to very similar values at the higher test temperatures (figure 26*b*) where effective lubricant penetration into the contact is facilitated (irrespective of the covered width) by the low lubricant viscosity.

Figure 26*b* shows that for the 160 mm contacts, the wear rate exhibits a reduction with increasing lubricant temperature (and thus decreasing lubricant viscosity) at the higher temperatures. For the two smaller displacement amplitudes, this effect can be attributed to more effective lubricant penetration into the contact (because a large covered width exists in both cases). However, for the experiments conducted with a 300 µm displacement amplitude (where the initial covered width is zero), the reduction in wear rate with increasing temperature at the higher temperatures cannot easily be attributed to enhanced lubricant penetration (it is suggested that this is a chemical effect, as outlined in §4.5). For experiments conducted with a 25 µm displacement amplitude (with the highest covered width), the decrease in wear rate with increasing temperature is monotonic, but for the other displacement amplitudes, the wear rate is observed to increase with increasing temperature over the lower temperature range (before decreasing again at the higher test temperatures). This increase in wear rate with increasing temperature (at the lower temperatures) indicates that there is another surface protection mechanism in operation (because an increase in temperature in and of itself increases the ease of lubricant penetration into the contact). It is also notable that the shift in behaviour from that at lower temperature (where the wear rates increase with test temperature) to the behaviour observed at the higher temperatures (where the wear rates decrease with increasing temperature) itself moves to higher temperature as the displacement amplitude increases (figure 26*b*).

Figure 26 also shows that for the less conforming contacts (with the 6 mm radius cylinders), similar behaviour (namely a rise in wear rate with increasing temperature at the lower test temperatures and then a reduction with temperature at the higher temperatures) is observed. Notably, the wear rates appear to be independent of displacement amplitude across the test temperatures examined. With a displacement amplitude of 25 µm, there is a small covered width, but with a 100 µm displacement amplitude, the covered width is zero. A similar decrease in wear rate with increasing temperature is observed at the highest temperature with the more-conforming contact (160 mm radius cylinder) with the 300 µm displacement amplitude where, again, the covered width is zero (figure 26*b*). In all these cases, because the covered width is either very small or zero, lubricant readily accesses all areas of the contact, and thus the decrease in wear rate as the temperature is increased from 150 to 230°C cannot be attributed to any enhancement in lubricant penetration which might be afforded by the commensurate reduction in lubricant viscosity.

### 4.3. Viscosity and velocity

**Figure 27.**
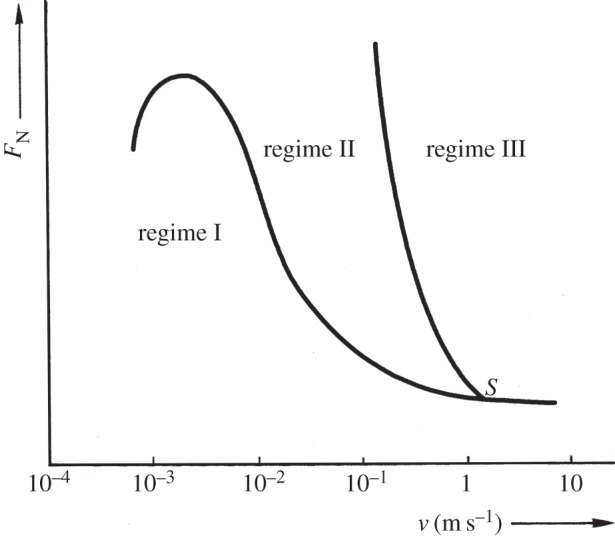
The IRG diagram describing regimes of behaviour for point contacts in terms of applied load and contact velocity (after [32]). Regime I represents partial elastohydrodynamic lubrication; regime II represents boundary lubrication; regime III represents virtually unlubricated contact (scuffing). Representative COFs in regimes I, II and III are 0.04–0.1, 0.2–0.4 and 0.3–0.5, respectively [35].

The least-conforming contacts (with 6 mm radius cylinders) tested at ambient temperature exhibited only a very small amount of wear and damage, with this occurring at high points on the contact; the lubricant film was clearly protective under these conditions. The IRG failure criteria map has been used to relate the observed wear behaviour with the concept of a protective lubricant film. Such maps have been developed for concentrated, highly loaded, lubricated contacts and an example of such a map is presented in figure 27. Lubricated wear in *regime I* is characterized by a low COF, and the contact is described as being within *partial* elastohydrodynamic lubrication (EHL) [32]; it is notable that this mode is operative for very low sliding speeds, covering the range of velocities observed in this work (figure 7). In partial EHL, only some parts of the contact touch and a portion of the load is carried by the lubricant film. Contacts that are in *regime I* typically have COF plots that reach a brief maximum of around approximately 0.1–0.2 and fall to a steady-state COF of approximately 0.1 [33]. This behaviour is very similar to that observed for the 6 mm contact fretted at ambient temperature for both displacement amplitudes examined (figures 13*b* and 16*b*). The IRG diagram indicates that a contact moves from partial EHL to more damaging regimes of behaviour (boundary lubrication in *regime II* and unlubricated scuffing in *regime III*) as the sliding velocity increases. It has been argued that there are two competing effects associated with the sliding speed; as the speed increases, the elastohydrodynamics will tend to increase the support offered by the film, but the frictional power dissipated in the contact tends to result in an increase in temperature which lowers the lubricant viscosity, which in turn results in a reduction in film support. The second effect is seen to dominate, and as such, the increase in sliding velocity leads to increased damage [33]. Czichos & Kirschke [34] demonstrated that the *regime I/regime II* boundary is temperature dependent, and that contacts will move to more damaging regimes of behaviour at lower loads and lower sliding velocities as the temperature is increased. Thus, an increase in temperature alone (with the load and sliding velocity remaining constant) may change the contact behaviour from partial EHL to a more damaging mode, with COF values typically being in the range 0.3–0.4 in this case. A similar increase in damage (figure 26*a*) and COF (figures 13*b* and 16*b*) with increasing temperature is observed for all the experiments conducted where lubricant penetration into the contact was effective at the lowest temperature.

In this work, because the fretting frequency is constant, an increase in displacement amplitude also results in an increase in velocity (figure 7 indicates sliding speeds of approximately 5 mm s^−1^ when Δ* = 25 µm, with values of 15–19 mm s^−1^ when Δ* = 100 µm). However, in contacts where the lubricant penetration is independent of fretting displacement, there is little dependence of damage on displacement (and thus sliding velocity) across the range of temperatures examined; this may result from the fact that in this case, while the frictional power dissipated increases with fretting displacement, the area over which the power is dissipated also increases, and the frictional power density (which will control temperature) is thus little changed.

### 4.4. Pressure

The IRG diagram in figure 27 indicates that a contact may move from partial EHL (*regime I*) to boundary lubrication (*regime II*) and then to scuffing (*regime III*) as the applied load is increased. In the work reported in this paper, the applied load was maintained at a constant value, but the changes in the contact conformity resulted in changes in nominal contact pressure from 386 to 75 MPa for the least and most conforming contacts, respectively. Despite this change in nominal contact pressure, in all cases examined, there is very little change in the wear rates across the temperature range for situations where lubricant penetration is good. This can be seen by comparing the wear rates for the least-conforming (6 mm radius cylinder) contacts at displacement amplitudes of 25 and 100 µm (figure 26*a*) and the most conforming (160 mm radius cylinder) contact at a displacement amplitude of 300 µm (figure 26*b*).

**Figure 28.**
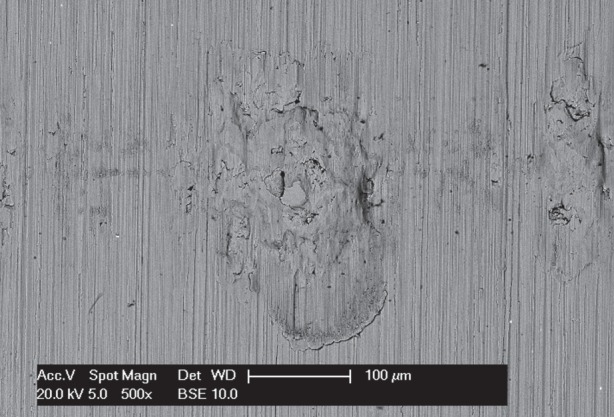
BSE image of the top surface of a wear scar on the flat specimen at high magnification, following an experiment conducted at ambient temperature, using a 6 mm cylinder with Δ* = 25 µm and *P* = 650 N.

In the light of this, an additional experiment was conducted with a contact with a 6 mm radius cylinder under a higher load (650 N) to assess whether the contact could be moved into *regime III* (scuffing) where significant surface damage could be expected. Figure 28 is a BSE image of the surface of the flat specimen following fretting at ambient temperature against a 6 mm radius cylinder with a 25 µm displacement amplitude under a 650 N load. In comparing this with the equivalent fretted surface following testing under the standard 250 N load (figure 14*e*), it can be seen that the increased load has indeed shifted the regime from a protective mode at the lower load to one involving adhesion (scuffing) at the higher load.

### 4.5. Chemical effects

For all of the geometries and displacement amplitudes examined, as the temperature was increased from 150 to 230°C, the wear rate was observed to *decrease*, and this decrease cannot be attributed in all cases to other phenomena previously discussed. However, there are changes in the chemistry of the system that can account for the behaviour observed. The lubricant in this research contains TCP as an EP additive, which is designed to protect the surface in conditions where a lubricant film itself is not offering protection. Such additives are activated by the high temperatures associated with asperity contact, but can also be activated during operation at elevated temperatures where the EP additive is designed to deliver reduced rates of wear in situations where the lubricant viscosity is becoming too small to offer fluid film support [36].

In addition, it has been noted by Wright [8] that oxygen diffusion through the lubricant film into the contact is restricted (but not eliminated), and that diffusion is enhanced by reductions in lubricant viscosity. Enhanced diffusion of oxygen as well as increased oxidation rates at the higher temperatures may result in the formation of oxides in the contact, which serve to protect the surfaces and reduce wear. Finally, lubricants are known to break down at higher temperatures, and may result in carbon being deposited on contacting surfaces. Large carbon-rich deposits have been observed in this work (e.g. figure 23) in situations where severe adhesion has occurred (severe adhesion results in high levels of power dissipation over small areas and thus elevated temperatures). However, it is feasible that carbon-rich material is being deposited within the contacts at elevated temperatures, and that this again serves to protect the surfaces from further damage.

## 5. Conclusion

The tribological behaviour of fretting contacts in lubricated conditions has been shown to be strongly dependent upon experimental parameters that influence the ability of (i) the lubricant to penetrate the contact and (ii) the lubricant to protect the contact (assuming that it has penetrated the contact). Lubricant penetration into the fretting contact is influenced by two primary physical parameters, namely (i) the covered width of the contact and (ii) the viscosity of the lubricant. The protection of the contact offered by the lubricant is related to four primary physical parameters, namely (i) lubricant viscosity, (ii) sliding velocity, (iii) nominal contact pressure, and (iv) chemical effects. The experimental parameters examined in this research (namely contact conformity, test temperature and fretting slip displacement) influence these physical parameters and in certain cases will compete, resulting in complex wear behaviour.

An increase in contact conformity under a constant normal load (by increasing the cylinder radius) will decrease the nominal contact pressure; this reduction in pressure has been shown to enhance the protection offered by the lubricant film. However, as the contact becomes more conforming, the covered width may also increase substantially (depending upon the slip amplitude). The increase in covered width results in an exclusion of lubricant from the central zone of the contact; the lubricant also effectively limits the ingress of oxygen into the contact and these together result in a substantial increase in wear and transfer rates in fretting. Lubricant penetration into the fretting contact is also influenced by displacement amplitude; higher displacement amplitudes result in smaller covered widths and therefore offer increased lubricant penetration. Temperature was observed to exert a complex influence on the lubricated fretting wear behaviour. An increase in temperature led to a reduction in the lubricant viscosity, which encouraged the lubricant to penetrate the contact and therefore limit wear.

While an increase in temperature has been shown to aid lubricant penetration into the contact, it will also weaken the support offered by any lubricant that has penetrated the contact. For contacts that exhibit sufficient lubricant penetration irrespective of lubricant viscosity, the wear rates are almost identical at each temperature, and exhibit an increase in wear rate as the temperature is increased from ambient temperature to 150°C. However, (for these contacts with sufficient lubricant penetration) a further increase to 230°C resulted in a decrease in the wear rate, indicating the influence of an additional effect associated with the chemistry and transport properties of the lubricants in the contact. The lubricant used in this study contains EP additives, which are activated at high temperatures and will protect the contact. Also, the diffusion of oxygen through the lubricant will increase with increasing temperature, leading to the possibility of formation of oxide films on the nascent fretted surfaces (which has generally been restricted by the presence of the lubricant film); even a thin layer of oxide will provide some barriers to intimate metallic contact between the two specimens and thus reduce severe adhesive transfer. Also, it is known that the lubricant breaks down at the highest test temperatures (carbon-rich deposits were found on the contacts that were fretted at the higher temperatures) and such carbon-rich deposits may act in the same way as any thin oxide films formed by providing some barrier to intimate metallic contact between the two specimens.

The IRG diagram gives some indication of the effect of velocity on contact conditions at velocities as low as those observed in fretting. It indicates that the primary effect of increasing velocity is to increase the temperature in the contact that reduces lubricant viscosity and moves the contact from partial EHL (low wear) to scuffing (higher wear). Because the fretting frequency was held constant in these tests, an increase in displacement amplitude resulted in a higher contact velocity. However, contacts that exhibit sufficient lubricant penetration (low conformity and higher displacement amplitudes) do not show any change in wear behaviour as the velocity is increased across the full range of temperatures examined. Although the dissipated frictional power would increase at higher displacement amplitudes, the frictional power density that controls the contact temperature is not changed significantly, because the fretting displacement has also increased.

Finally, contact pressure is itself also found to influence the fretting wear behaviour of lubricated fretting contacts where lubricant penetration into the contact is sufficient; for example, a transition between the lubricant film being protective at lower loads to the film being broken down resulting in severe metallic transfer under higher loads was observed.

## Supplementary Material

Fig-8a. Each file has a figure number which clearly relates the data to the figure as presented.

## Supplementary Material

Fig-8b

## Supplementary Material

Fig-10a

## Supplementary Material

Fig-10b

## Supplementary Material

Fig-13a

## Supplementary Material

Fig-13b

## Supplementary Material

Fig-16a

## Supplementary Material

Fig-16b

## Supplementary Material

Fig-19a

## Supplementary Material

Fig-19b

## Supplementary Material

Fig-22a

## Supplementary Material

Fig-22b

## Supplementary Material

Fig-26a

## Supplementary Material

Fig-26b

## Data accessibility

The datasets supporting this article have been uploaded as part of the electronic supplementary material.
